# Nutrient-Poor Breeding Substrates of Ambrosia Beetles Are Enriched With Biologically Important Elements

**DOI:** 10.3389/fmicb.2021.664542

**Published:** 2021-04-26

**Authors:** Maximilian Lehenberger, Nina Foh, Axel Göttlein, Diana Six, Peter H. W. Biedermann

**Affiliations:** ^1^Research Group Insect-Fungus Symbiosis, Department of Animal Ecology and Tropical Biology, University of Würzburg, Würzburg, Germany; ^2^Chair of Forest Entomology and Protection, University of Freiburg, Freiburg im Breisgau, Germany; ^3^Center for Medical Physics and Engineering, Max Schaldach Endowed Professorship for Biomedical Engineering, Friedrich-Alexander University Erlangen-Nürnberg (FAU), Erlangen, Germany; ^4^Forest Nutrition and Water Resources, TUM School of Life Sciences Weihenstephan, Technical University of Munich, Freising, Germany; ^5^Department of Ecosystem and Conservation Sciences, W. A. Franke College of Forestry and Conservation, University of Montana, Missoula, MT, United States

**Keywords:** ambrosia beetle, Ecological stoichiometry, microbiome, nutrients, macro- and micro-elements, element translocation

## Abstract

Fungus-farming within galleries in the xylem of trees has evolved independently in at least twelve lineages of weevils (Curculionidae: Scolytinae, Platypodinae) and one lineage of ship-timber beetles (Lymexylidae). Jointly these are termed ambrosia beetles because they actively cultivate nutritional “ambrosia fungi” as their main source of food. The beetles are obligately dependent on their ambrosia fungi as they provide them a broad range of essential nutrients ensuring their survival in an extremely nutrient-poor environment. While xylem is rich in carbon (C) and hydrogen (H), various elements essential for fungal and beetle growth, such as nitrogen (N), phosphorus (P), sulfur (S), potassium (K), calcium (Ca), magnesium (Mg), and manganese (Mn) are extremely low in concentration. Currently it remains untested how both ambrosia beetles and their fungi meet their nutritional requirements in this habitat. Here, we aimed to determine for the first time if galleries of ambrosia beetles are generally enriched with elements that are rare in uncolonized xylem tissue and whether these nutrients are translocated to the galleries from the xylem by the fungal associates. To do so, we examined natural galleries of three ambrosia beetle species from three independently evolved farming lineages, *Xyleborinus saxesenii* (Scolytinae: Xyleborini), *Trypodendron lineatum* (Scolytinae: Xyloterini) and *Elateroides dermestoides* (Lymexylidae), that cultivate unrelated ambrosia fungi in the ascomycete orders Ophiostomatales, Microascales, and Saccharomycetales, respectively. Several elements, in particular Ca, N, P, K, Mg, Mn, and S, were present in high concentrations within the beetles’ galleries but available in only very low concentrations in the surrounding xylem. The concentration of elements was generally highest with *X. saxesenii*, followed by *T. lineatum* and *E. dermestoides*, which positively correlates with the degree of sociality and productivity of brood per gallery. We propose that the ambrosia fungal mutualists are translocating essential elements through their hyphae from the xylem to fruiting structures they form on gallery walls. Moreover, the extremely strong enrichment observed suggests recycling of these elements from the feces of the insects, where bacteria and yeasts might play a role.

## Introduction

In 1836, the monk Josef Schmidberger noticed a whitish layer in tunnel systems of some scolytine beetles within the sap- and heartwood (i.e., xylem) of his apple trees (Schmidberger, 1836). Observing the beetles in more detail he realized that this layer was nutritional and – in his view – was produced by the trees to nurture the beetles. Therefore, he termed this layer “ambrosia” (after the mythological perfect food of the gods). This layer was later found to be fungal fruiting structures (Hartig, 1844) and their beneficiaries came to be called “ambrosia beetles.” Even then it was regarded rather exceptional for insects to live within xylem, which is now known to be chemically well defended, nutritionally poor and extremely biased toward certain elements, particularly carbon (C), hydrogen (H), and oxygen (O) (Filipiak and Weiner, 2014; Pretzsch et al., 2014).

While most bark beetles (*sensu lato*) (Curculionidae: Scolytinae) colonizing the phloem or xylem of trees are well known for their facultative mutualism with fungi, ambrosia beetles (*sensu stricto*) among them have long been known for their obligate mutualisms with fungal symbionts (Kirkendall et al., 2015; Biedermann and Vega, 2020). In a broad sense, the term “ambrosia beetle” is used for all xylem-boring and fungus-farming beetles in the curculionidae subfamilies Scolytinae (bark beetles) and Platypodinae (pinhole borers) as well as the Lymexylidae (ship-timber beetles) (Neger, 1908a). Ambrosia beetles are thus a polyphyletic group with at least 13 evolutionary independent origins of fungus farming (Biedermann and Vega, 2020). Common to all of them is their species-specific, obligate nutritional mutualism with filamentous fungi, which they transmit vertically in mycetangia (extracuticular spore-carrying organs; see Francke-Grosmann, 1956) and more or less actively farm in often social family groups (but Lymexylidae are solitary; Francke-Grosmann, 1967; Beaver, 1989; Kirkendall et al., 2015; Hulcr and Stelinski, 2017; Biedermann and Vega, 2020). Their mutualistic fungi are from at least five ascomycete (Ophiostomataceae, Ceratocystidaceae, Nectriaceae, Bionectriaceae, Saccharomycetaceae) and two basidiomycete (Peniophoraceae, Meruliaceae) (Hulcr and Stelinski, 2017; Biedermann and Vega, 2020) families. These species-specific mutualistic associates are highly adapted to their ambrosia beetle host. They strongly depend on the beetle for dispersal, are not capable of surviving outside the mutualism, produce nutrient-rich fruiting structures within the beetles’ galleries, and are the sole or at least major food source for the beetles (Neger,1908a,b; Batra, 1963, 1966, 1967, 1973, 1985; Francke-Grosmann, 1966, 1967; Batra, 1967).

All herbivorous animals are confronted with a stoichiometric mismatch between the concentrations of elements required for growth and survival including nitrogen (N), phosphorus (P), potassium (K), calcium (Ca), magnesium (Mg), iron (Fe), zinc (Zn), manganese (Mn), copper (Cu) and sodium (Na), and those found in their plant diet, which is rich in only C, H, and O (Sterner and Elser, 2002). For insects feeding within xylem this mismatch is extreme, as 99% of this tissue typically consists of C, H, and O (Fengel and Wegener, 1989; Filipiak and Weiner, 2014) and elements such as N or P must be enriched by factors between 100 and 10,000 to support insect growth (Six and Elser, 2020). Hence, they have only two options (or a combination of both); one is to develop very slowly and process massive amounts of plant material (see e.g., cerambycid or buprestid beetles) while the other is to supplement the diet with ectosymbiotic fungi that grow into the wood, and translocate and concentrate required elements for growth (Buchner, 1928; Martin and Kukor, 1984; Filipiak and Weiner, 2014). The latter process is assumed for ambrosia beetles that feed on their mutualistic ambrosia fungi (Biedermann and Vega, 2020), but whether rare elements are indeed absorbed by the fungi and translocated to the ambrosia layers made of asexual fruiting structures on the gallery walls has never been examined.

Ecological stoichiometry (hereafter termed ES) is a powerful approach which has the potential to answer such questions. In general, ES describes the movement and balance of elements in natural systems and how this is influenced by ecological interactions and processes (Sterner and Elser, 2002). Basically, ES offers the opportunity to examine the availability and limitations of elements within natural systems and thus, is a useful tool to gain insights into tropic relations such as the ecology and function of insect-microbe mutualisms. Nevertheless, only very few studies applied ES to these systems (Six and Elser, 2019, 2020). Six and Elser (2019) examined for the very first time within a bark beetle species, how fungal mutualists associated with *Dendroctonus brevicomis* concentrate and translocate elements such as P and N from the sapwood and phloem to the bark, where the beetles live and feed. To date, almost no studies applying ES to fungus-farming insect systems, such as ambrosia beetles, have been conducted and there have only been a few studies dealing with the nutrient flow in plant-fungus or animal-animal mutualisms (Kay et al., 2004; Johnson, 2010; Mariotte et al., 2017). One of the few exceptions focused on a fungus-farming termite, *Odontotermes formosanus*, showing that certain elements (such as K, Mg, Mn, and Ca and some others) are highly enriched within the symbiotic fungus as well as within nest soil (see Li et al., 2012).

All organisms require a number of macro- and micro-nutrients to grow, develop, and reproduce. Insects feeding in plant tissues like wood are especially challenged to acquire the nutrients they need and most, if not all, have symbioses with microbes that aid in alleviating imbalances (Buchner, 1928). For instance, N (needed for producing proteins and amino acids), S (needed for production of the amino acid methionine and cysteine), and P (needed for production of ribosomes, RNA, DNA, lipid layers, and ATP) (McDowell, 2003) occur in wood in very low concentrations (Fengel and Wegener, 1989; Filipiak et al., 2016). So wood-dwelling insects often make use of specific microbes to enrich their diet (Täyasu et al., 1994; Ayayee et al., 2014; Ulyshen, 2015; Filipiak et al., 2016; Filipiak and Weiner, 2017). Filamentous fungi among them may acquire the rare nutrients by growing far into plant tissues (Six and Elser, 2019), whereas some bacteria are capable of fixing atmospheric nitrogen (Peklo and Satava, 1949; Bridges, 1981; Täyasu et al., 1994; Six, 2003; Bleiker and Six, 2014; Filipiak and Weiner, 2014; Filipiak et al., 2016). Since ambrosia beetles do not feed outside their galleries, all nutrients needed for growth and reproduction for themselves and for their fungi must come from the surrounding xylem or the atmosphere (only N).

Here, we examined the availability and possible translocation of critical nutrients by fungi for three well-known and widespread ambrosia beetle species in three independently evolved mutualisms of Xyleborini (Curculionidae: Scolytinae) with *Raffaelea* (Ophiostomatales: Ophiostomataceae) fungi, Xyloterini (Curculionidae: Scolytinae) with *Phialophoropsis* (Microascales: Ceratocystidaceae) fungi and Lymexylidae with *Alloascoidea* (Saccharomycetales: Saccharomycetaceae) filamentous yeasts: (i) The fruit-tree pinhole borer *Xyleborinus saxesenii* (Ratzeburg) (Xyleborini), (ii) the striped ambrosia beetle *Trypodendron lineatum* (Olivier) (Xyloterini), and (iii) the ship-timber beetle *Elateroides dermestoides* (syn. *Hylecoetus dermestoides*) (Linnaeus) (Lymexylidae). All three beetles are native to Europe but differ strongly in their social systems and farming behaviors. *Xyleborinus saxesenii* is a cooperatively breeding species with a chamber-like gallery, where brood of all stages are found together with adult helpers, adult reproductives (mothers) and the farmed ambrosia fungi *Raffaelea sulphurea* (L.R. Batra) T.C. Harrington and *R. canadensis* (L.R. Batra) T.C. Harrington (Biedermann and Taborsky, 2011; Biedermann et al., 2013). This beetle shows little host preference, exhibits alloparental brood care and brother-sister inbreeding and can colonize the natal nest for more than two generations (Biedermann, 2010). *Trypodendron lineatum* prefers conifers and exhibits bi-parental care, but larvae develop within separate cradles with tunnel walls covered by *Phialophoropsis ferruginea* (Mathiesen-Käärik, 1953; Lehenberger et al., 2019; Mayers et al., 2020). Their galleries are active for only a single generation. No parent-offspring contact occurs in the host tree-generalist *E. dermestoides* (Coleoptera: Lymexylidae) whose larvae bore solitary tunnels and farm the yeast-like *Alloascoidea hylecoeti* (Neger) L.R. Batra (Francke-Grosmann, 1952; Batra and Francke-Grosmann, 1961; Batra, 1967). Developmental periods range from 1 to 2 months for the two scolytine species to 2–3 years for the lymexylid beetles (Francke-Grosmann, 1952; Popo and Thalenhorst, 1974; Biedermann et al., 2009). Differences in development may be partly explained by body size (*X. saxesenii* ∼2 mm, *T. lineatum* ∼3,5 mm, *E. dermestoides* 6–19 mm).

Here, we aimed to investigate if galleries of all three species are generally enriched with certain elements compared to uncolonized xylem indicating that the fungi may translocate and concentrate crucial nutritional elements from the surrounding xylem. We first visualized the elemental composition of xylem and gallery walls using scanning electron microscopy coupled with energy dispersive X-ray analysis (SEM-EDX). This technique has, to our knowledge, never been applied to an animal-microbe mutualism. It visualizes elements on biological surfaces but does not allow exact elemental quantification of such samples. Therefore, we then quantified the elemental composition of both galleries and xylem using acid digestion and inductively coupled plasma optical emission spectroscopy (ICP-OES) which is widely used for examining woody samples (e.g., Huber et al., 2004; Górecka et al., 2006; Weis et al., 2009; Wellinger et al., 2012).

## Materials and Methods

### Collection and Preparation of Beetle Galleries

We collected galleries of *X. saxesenii* (*N* = 13) and *E. dermestoides* (*N* = 1) in May 2019 from freshly dead beech (*Fagus sylvatica*) in the Bavarian Forest National Park (Neuschönau, Germany). Galleries of *T. lineatum* were collected from Norway spruce (*Picea abies*) in June 2019 in Mitterberg, Austria (*N* = 10). The galleries of the first two species still contained living insects, whereas *T. lineatum* galleries were from the previous season, but stored dry until dissection. For dissection of individual galleries, we used a chainsaw, an axe, and a spade chisel. Adult and immature specimen were either knocked out of the gallery or collected with fine tweezers. Immediately after opening a gallery, we carefully collected three different types of samples (each approx. 1 cm × 1 cm and 2–3 mm deep) for elemental analysis: (i) gallery, (ii) surrounding xylem (about 1 cm next to the gallery in direction of wood fibers), and (iii) control xylem (at least 5 cm away from the gallery perpendicular to the direction of wood). Samples were stored in glass vials and galleries (for SEM-EDX) in paper bags at −20°C until they were processed.

To dry samples, we transferred them to a glass-desiccator filled with silica gel (without contact to the samples; Roth, Germany) and dried them for approximately 3 months. Galleries used for the SEM-EDX analysis were oven-dried for 1 week at 50°C within a slightly opened paper-bag ensuring the protection from any contamination and then stored within a glass-desiccator until they were analyzed.

### Semi-Quantification and Visualization of Elements by Energy-Dispersive X-ray Scanning-Electron-Microscopy (SEM-EDX)

Scanning electron microscopy coupled with energy dispersive X-ray analysis provides a semi-quantitative view of elemental compositions on the surface of samples (to a depth of ∼2–3 μm). It detects and identifies elements on the surface layer of a sample by detecting released element-specific radiation energy (e.g., but see also Hudgins et al., 2003; De Vetter et al., 2006; Shindo and Oikawa, 2013; Wang and Dibdiakova, 2014). However, to receive reliable data, it is important to minimize irregularities on the surface of an analyzed sample. Our flat sectioning of galleries reduced irregularities and allowed us to use it as a basis for the choice of elements to focus for the following ICP-OES absolute quantification. Three galleries from *X. saxesenii*, two from *T. lineatum* and one from *E. dermestoides* were used for SEM-EDX. With this design we aimed to test if certain elements are enriched within the beetles’ galleries compared to the surrounding xylem and whether this is influenced by the direction of the fibers (fungi often grow along the wood fibers).

To prepare samples, we carefully opened each dried gallery and cut it to a size of 10 cm × 10 cm to fit onto an aluminum plate. To fix the position of each gallery on the plate, we used epoxy-resin (EPODEX, Germany). Afterward, we carefully removed loose material like sawdust, beetle feces, and loose fungal mycelium from the surface of each gallery using filter-cleaned compressed air.

An EVO MA15 (Zeiss; Software: Esprit Version) scanning electron microscope (SEM) connected to an energy dispersive X-ray detector (EDX; Detector: Bruker XFlash 5010; Software: Bruker Quantax Esprit 1.9) within a semi-vacuum atmosphere was used for the measurements applying a primary energy of 20 keV. Just before the measurements, we placed a few small pieces of aluminum foil onto our samples (outside the areas of interest), which helped us to navigate and locate the areas of interest under the SEM. As we introduced this element to all samples used for SEM-EDX, we did not include it in our further analyses. A single measurement covered an area of about 600 μm × 800 μm. Multiple measurements from each gallery were taken along two lines, one in a vertical and the other in a horizontal direction to the direction of the wood fibers, covering both the inside (“gallery”) as well as the outside (“surrounding xylem”) of the beetle’s gallery. For two measurements (one gallery each of *X. saxesenii* and of *T. lineatum*), we used the mapping function of the SEM-EDX, which allowed us to visualize the abundance of certain elements using different colors. Each gallery was exposed to an electron irradiation (20 keV) for approximately 10 min, in which we allowed the EDX-detector to identify element-specific radiation energies that were released by the samples.

Finally, we created line charts to visualize the presence of certain elements using ggplot in R (version 1.2.5033) with the packages “ggplot2” (Wickham, 2016) and “plotly” (Sievert, 2020). Colored lines (one color per element) were manually added and indicate the respective elements, while the length of each vertical line approximates the size of each peak (i.e., abundance of the element) with the exception of C, N, and O which were either overabundant (C, O) or poorly quantifiable (N) using SEM-EDX.

### Exact Quantification of Elements Using Inductively Coupled Plasma Optic Emission Spectroscopy (ICP-OES) and an Element Analyzer

Here, we examined galleries from *T. lineatum* (*N* = 7–8; see Supplementary Table 1) and *X. saxesenii* (*N* = 9–10; see Supplementary Table 2) using acid digestion and ICP-OES (ARCOS by Spectro, Kleve, Germany), while the C, N, and H concentrations were determined by dry combustion in an element analyzer (*T. lineatum*: *N* = 5–8, see Supplementary Table 1; *X. saxesenii*: *N* = 9–10, see Supplementary Table 2) (vario EL III by Elementar, Langenselbold, Germany). The variation in the number of replicates is caused by few samples of insufficient size as well as by some extreme outliers (i.e., measurement errors, see below) which were removed prior to analyses.

The quantification was accomplished at the Professorship of Forest Nutrition and Water Resources (Technical University of Munich, Freising, Germany). The dried samples were analyzed for their elemental composition according to German Forest Analytical Standards (König et al., 2005). Based on the received output of the SEM-EDX analysis, we quantified the following elements: P, K, S, Mg, Fe, Mn, Cu, Zn, Ca (after acid digestion using ICP-OES) as well as C, H, and N (using an element analyzer). Approximately 60 mg per woody sample were digested with freshly distilled nitric acid (HNO3) at 160°C for 10 h within quartz vessels using a high-pressure digestion apparatus (Seiff, Germany). The digest was diluted to 14 ml with distilled water and analyzed for element concentration by ICP-OES.

Since analytically conspicuous samples could not be checked (each sample was unique and the analysis is destructive), extreme outliers were removed from the total data set according to Tukey’s procedure (Tukey, 1977). The concentration of each investigated element per individual treatment was visualized with ggplot – boxplots in R (version 1.2.5033) using the package “ggplot2” (Wickham, 2016). Additionally, linear models (sqrt transformed data) were conducted to detect significant differences between the three different treatments (Gallery, Surrounding xylem, Control xylem) for each individual beetle species. Here, we used the R-packages “LMERConvenienceFunctions” (CRAN.R-project.org/package = LMERConvenienceFunctions), “multcomp” (Hothorn et al., 2008), “lsmeans” (Lenth, 2016), and “lme4” (Bates et al., 2015). Further, we calculated enrichment ratios for each examined element per treatment relative to control xylem as well as the molar ratios for C:P (carbon:phosphorus), C:N (nitrogen), N:P, N:S (sulfur), N:Ca (calcium), N:K (potassium), N:Mg (magnesium), N:Mn (manganese), and N:Zn (zinc) for each of the three treatments.

## Results

### Semi-Quantification and Visualization of Elements by Energy-Dispersive X-ray Scanning-Electron-Microscopy (SEM-EDX)

**FIGURE 1:**
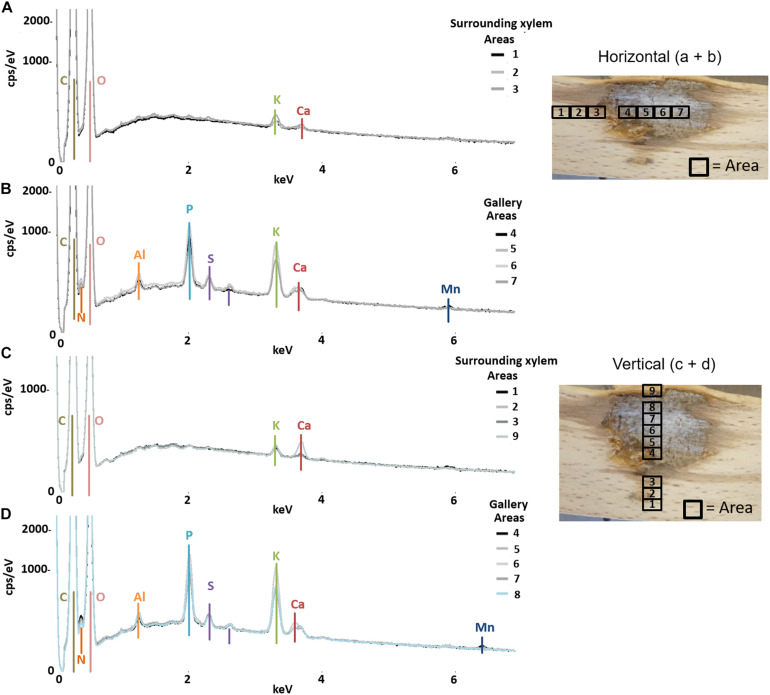
Elemental composition of gallery walls and xylem surrounding a gallery of *Xyleborinus saxesenii* (ID = X.sax13) using SEM-EDX. The gallery was examined in horizontal **(A,B)** and vertical **(C,D)** direction respective to wood grain; location and numbers of measured areas are given on the right. The *Y*-axis of each plot is counts per second () per electronvolt (eV) of each element, while the *X*-axis is the applied electricity in kilo-electronvolt (keV).

**FIGURE 2:**
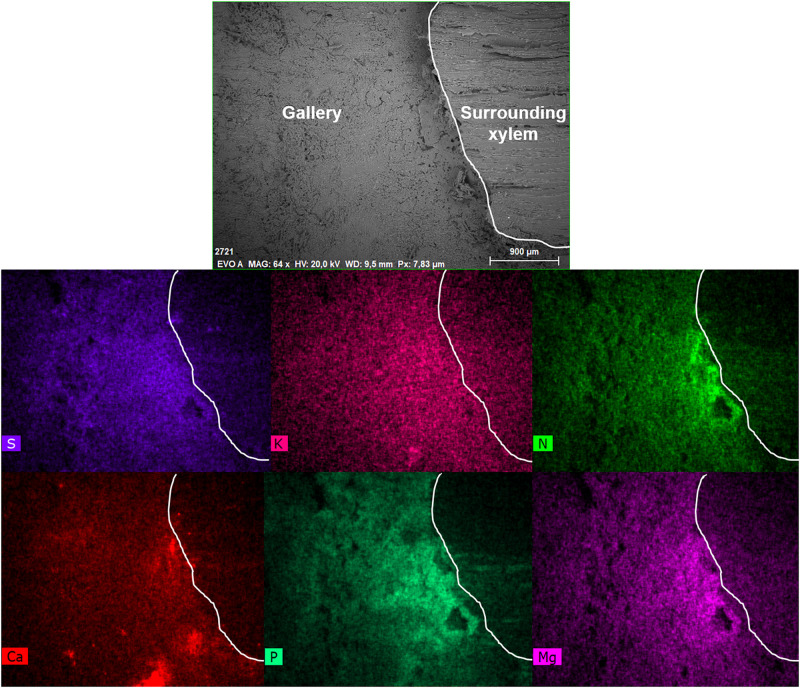
Visualization of elements within a gallery of *Xyleborinus saxesenii* (ID = X.sax2) using the mapping function of the SEM-EDX. The SEM image on top (EVO A) is the inside of the gallery (left) and the surrounding xylem (right, starting from the ridge). The border from gallery to surrounding xylem was indicated with a white line in each image. Scale bar and magnification (64×) are given at the bottom of the image. The intensity of the colors in the images below reflect the abundance of the elements sulfur (S), potassium (K), nitrogen (N), calcium (Ca), phosphorus (P), and magnesium (Mg).

**FIGURE 3:**
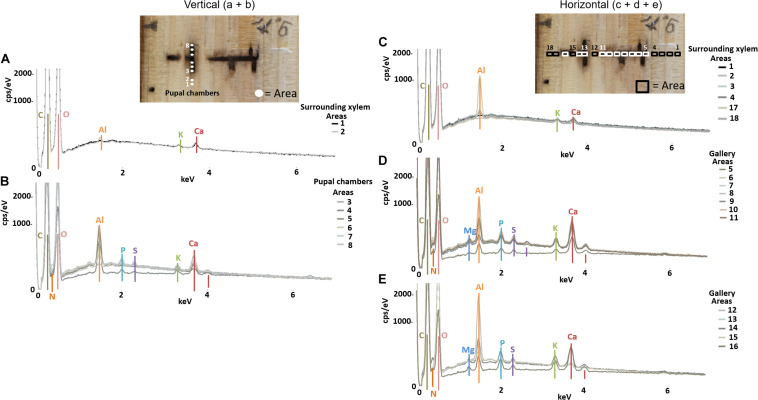
Elemental composition of gallery walls, pupal chambers and xylem surrounding a gallery of *Trypodendron lineatum* (ID = T.lin9) using SEM-EDX. Pupal chambers were examined in vertical **(A,B)** direction, while the gallery was examined in horizontal **(C–E)** direction, respective to wood grain; location and numbers of measured areas are given in the photos on top. The *Y*-axis of each plot is counts per second (cps) per electronvolt (eV) of each element, while the *X*-axis is the applied electricity in kilo-electronvolt (keV).

**FIGURE 4:**
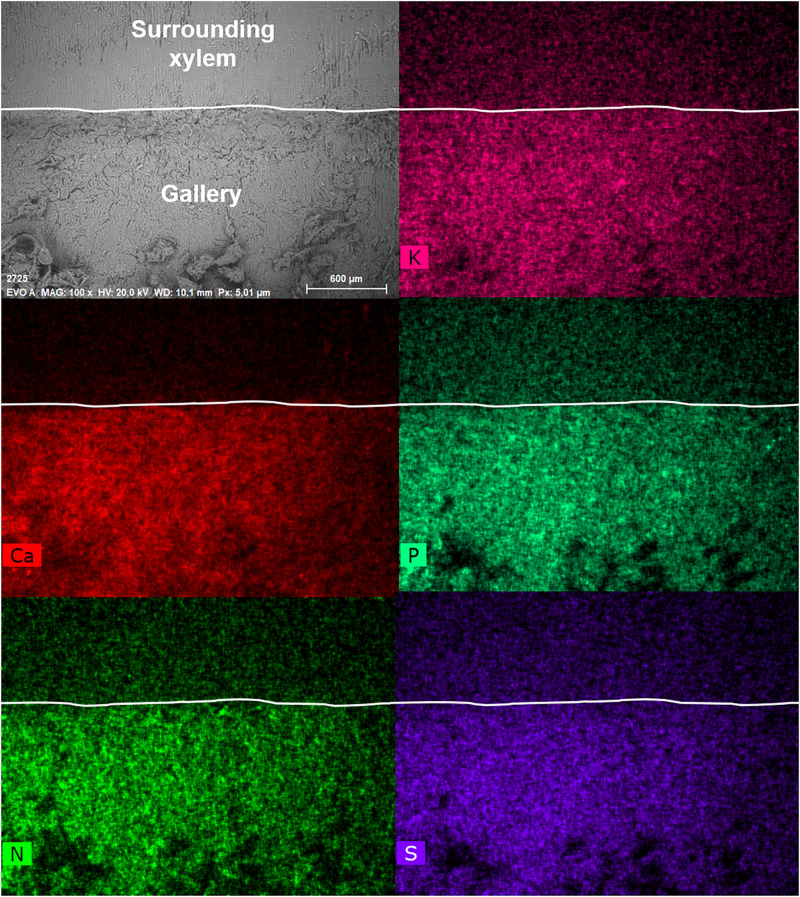
Visualization of elements within a gallery of *Trypodendron lineatum* (ID = T.lin9) using the mapping function of the SEM-EDX. The SEM image on the top left (EVO A) shows the inside of the gallery (bottom) and the surrounding xylem (top, starting from the ridge). The border from gallery to surrounding xylem was indicated with a white line in each image. Scale bar and magnification (100×) are given at the bottom of the image. The intensity of the colors in the other images reflect the abundance of the elements potassium (K), calcium (Ca), phosphorus (S), nitrogen (N), and sulfur (S).

**FIGURE 5:**
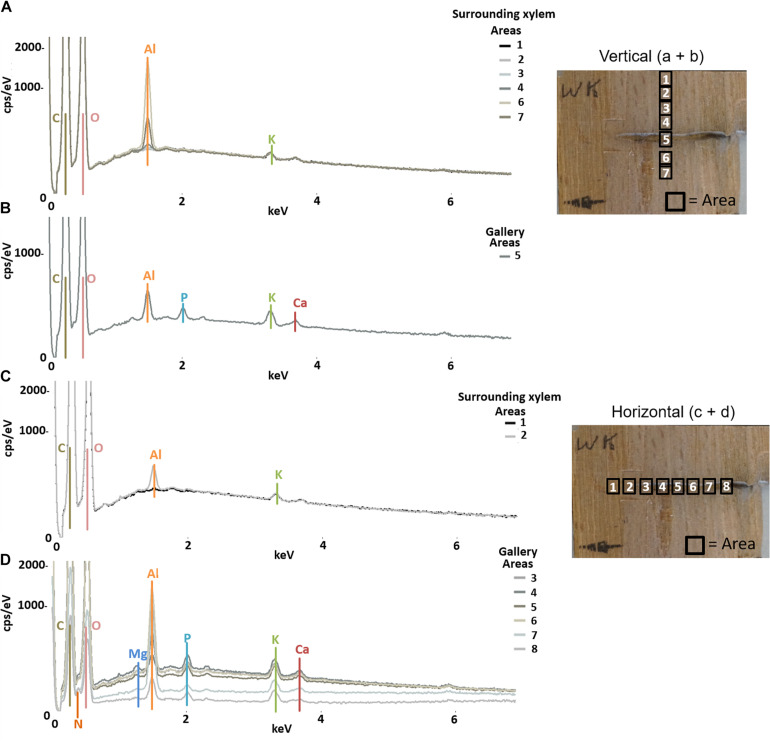
Elemental composition of gallery walls and xylem surrounding a gallery of *Elateroides dermestoides* (ID = HD) using SEM-EDX. The gallery was examined in vertical **(A,B)** and horizontal **(C,D)** direction respective to wood grain; location and numbers of measured areas are given in the photos on the right. The *Y*-axis of each plot is counts per second (cps) per electronvolt (eV) of each element, while the *X*-axis is the applied electricity in kilo-electronvolt (keV).

The semi-quantitative SEM-EDX analysis revealed that all examined galleries [3 × *X. saxesenii*, (Figures 1, 2 and Supplementary Figures 1, 2); 2 × *T. lineatum*, (Figures 3, 4 and Supplementary Figure 3); 1 × *E. dermestoides*, (Figure 5)] were enriched with N, Mg, P, S, K, and Ca (in case of *E. dermestoides*, S appears to be absent within the analyzed gallery). Further, Mg could not be identified within one of the three galleries of *X. saxesenii* (see Figure 1) using SEM-EDX, but the presences of this element within this specific gallery was proven by the EDX-Mapping function (see Figure 2). Samples from uncolonized xylem had very low elemental compositions, which seemed to be independent of the direction of the wood fibers (i.e., horizontal vs. vertical measurements from the gallery in Figures 1, 3, 5). Within uncolonized xylem, we mainly detected Ca, K, C, and O (for *E. dermestoides*, Ca could not be detected outside the gallery), which appears to be also present within the galleries of all examined beetles. C and O are very abundant in all types of samples, so we did not include them in the EDX-mapping. We detected Mn only within the analyzed galleries of *X. saxesenii*. For *T. lineatum*, we examined both galleries and pupal chambers. Here, pupal chambers (Figures 3A,C; Supplementary Figures 3A,B) seemed to be generally enriched with P, S, Ca, K, and N, while Mg appears to be absent. The EDX-Mapping function supported our findings as visualized gallery tissues were enriched with S, N, Ca, P, and Mg for *X. saxesenii* (see Figure 2) as well as with K, Ca, P, and N for *T. lineatum* (see Figure 4) relative to surrounding xylem. Raw data for the SEM-EDX analysis can be found in the supplementary material (see Supplementary Datas 1–3).

### Exact Quantification of Elements Using Inductively Coupled Plasma Optic Emission Spectroscopy (ICP-OES) and an Element Analyzer

**FIGURE 6:**
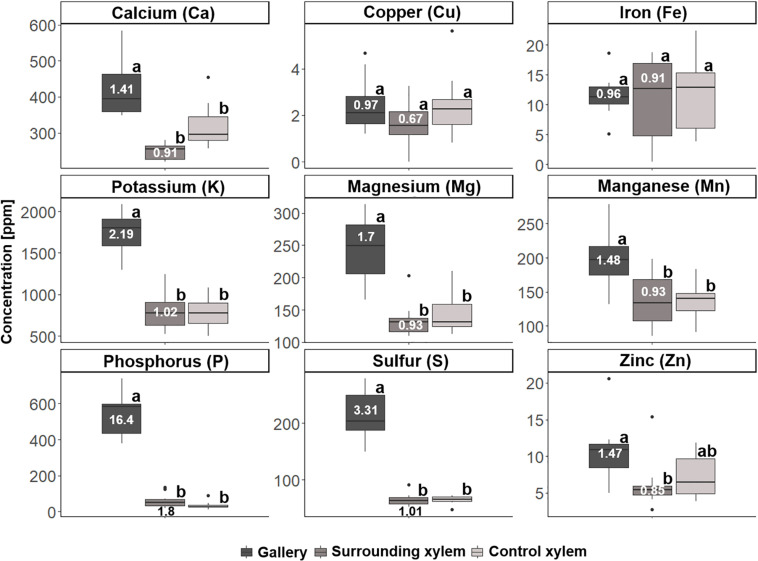
Concentrations of the different elements for the three types of samples taken from galleries of *Xyleborinus saxesenii* (*N* = 9–10 per group): (i) gallery, (ii) xylem next to gallery, and (iii) control xylem. Elemental abundances are given in parts per million (ppm). Different letters next to boxplots (a, b) indicate significant differences between the respective group per individual element. Enrichment ratios (white numbers) for treatment i and ii were added to boxplots for each element. For P and S, enrichment rations were indicated below the boxplot (black numbers).

**FIGURE 7:**
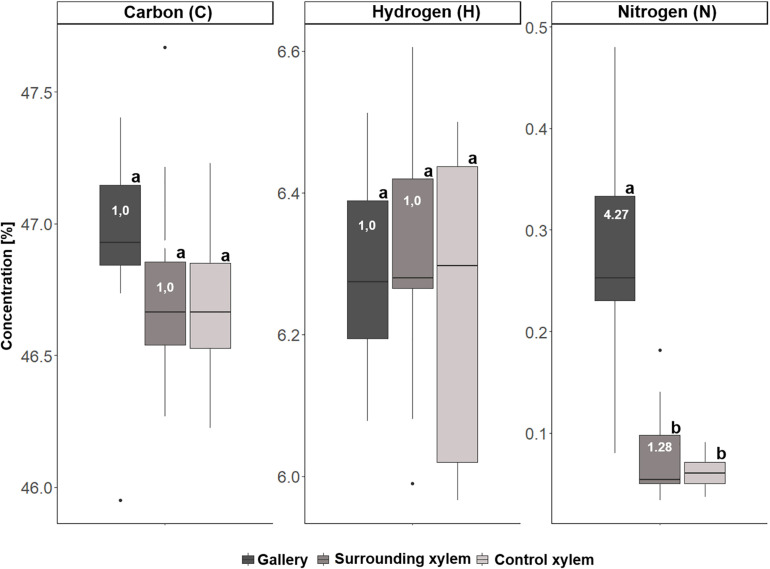
Concentrations of carbon (C), hydrogen (H), and nitrogen (N) for the three types of samples taken from *Xyleborinus saxesenii* (*N* = 9–10 per group): (i) gallery, (ii) xylem next to gallery, and (iii) control xylem. Different letters next to boxplots (a, b) indicate significant differences between the respective group per individual element. Enrichment ratios (white numbers) for treatment i and ii were added to boxplots for each element.

In a second step, we analyzed samples of 10 galleries of *X. saxesenii* and 8 galleries of *T. lineatum*, that were divided into the treatments (i) gallery, (ii) surrounding xylem, and (iii) control xylem. Generally, we confirmed our previous findings using quantitative ICP-OES and the element analyzer. We found significant incorporation of Ca (*p* = 0.0005, i to ii; *p* = 0.0141, i to iii), K, Mg, P, S, N (*p* < 0.0001, i to ii and iii), Mn (*p* = 0.0066, i to ii; *p* = 0.0087, i to iii), and Zn (*p* = 0.0173, i to ii) within galleries of *X. saxesenii* (see Figures 6, 7; Supplementary Table 1). Enrichment ratios of galleries relative to control xylem ranged from no enrichment of Fe (i: 0.96, ii: 0.91), Cu (i: 0.97, ii: 0.67), C (i: 1.0, ii: 1.0), and H (i: 1.0, ii: 1.0) to slight enrichment of Ca (i: 1.41, ii: 0.91), K (i: 2.19, ii: 1.02), Mg (i: 1.7, ii: 0.93), Mn (i: 1.48, ii: 1.01), and Zn (i: 1.47, ii: 0.85), and to strong enrichments of P (i: 16.4, ii: 1.8), S (i: 3.31, ii: 1.01), and N (i: 4.27, ii: 1.28) (Supplementary Table 3). These enrichment ratios were also reflected by the C:N, C:P, and N:P ratios as well as all other ratios (N:S, N:Ca, N:K, N:Mg, N:Mn, N:Zn) (see Supplementary Table 5).

**FIGURE 8:**
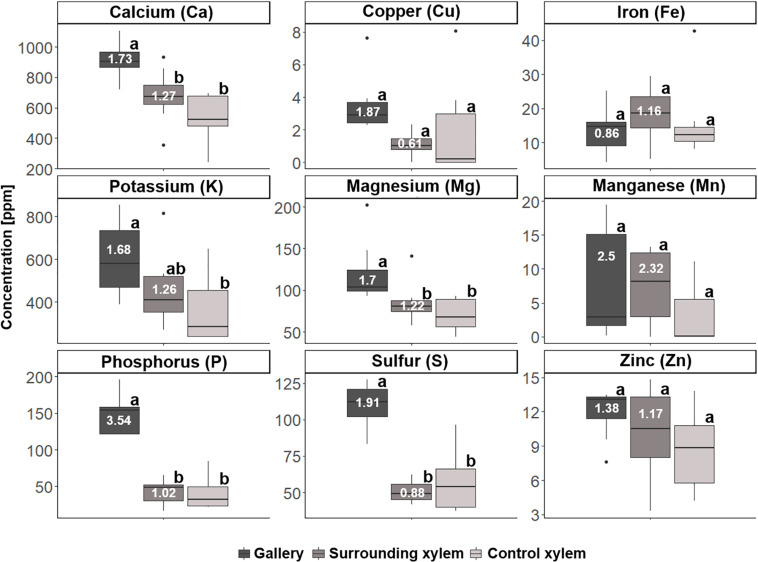
Concentrations of the different elements for the three types of samples taken from *Trypodendron lineatum* (*N* = 7–8 per group): (i) gallery, (ii) xylem next to gallery, and (iii) control xylem. Elemental abundances are given in parts per million (ppm). Different letters next to boxplots (a, b) indicate significant differences between the respective group per individual element. Enrichment ratios (white numbers) for treatment i and ii were added to boxplots for each element.

**FIGURE 9:**
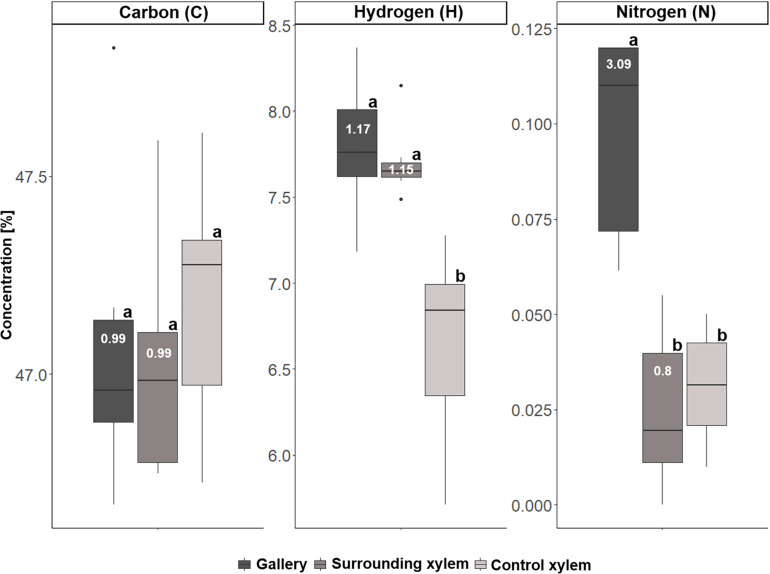
Concentrations of carbon (C), hydrogen (H), and nitrogen (N) the three types of samples taken from *Trypodendron lineatum* (*N* = 5–8 per group): (i) gallery, (ii) xylem next to gallery, and (iii) control xylem. Different letters next to boxplots (a, b) indicate significant differences between the respective group per individual element. Enrichment ratios (white numbers) for treatment i and ii were added to boxplots for each element.

For *T. lineatum*, we did not analyze pupal chambers, but focused on the beetles’ main galleries, which were enriched with Ca (*p* = 0.0281, i to ii; *p* = 0.0004, i to iii), K (*p* = 0.0149, i to iii), Mg (*p* = 0.0479, i to ii; *p* = 0.0026, i to iii), P, S (*p* < 0.0001, i to ii and iii), and N (*p* = 0.0005, i to ii; *p* = 0.0036, i to iii) (see Figures 8, 9; Supplementary Table 2). Interestingly, the amount of H (see Figure 9) was found to be higher in treatment i and ii compared to iii (*p* = 0.0003, i to iii; *p* = 0.0004, ii to iii). Enrichment ratios of galleries relative to control xylem ranged from no enrichment of Fe (i: 0.86, ii: 1.16) and C (i: 0.99, ii: 0.99), to slight enrichments of Ca (i: 1.73, ii: 1.27), Cu (i: 1.87, ii: 0.61), K (i: 1.68, ii: 1.26), Mg (i: 1.7, ii: 1.22), Mn (i: 2.5, ii: 2.32), S (i: 1.91, ii: 0.88), Zn (i: 1.38, ii: 1.17), and H (i: 1.17, ii: 1.15), and strong enrichments of P (i: 3.54, ii: 1.02) and N (i: 3.09, ii: 0.80) (Supplementary Table 4). Again enrichment ratios were well reflected by element ratios (C:N, C:P, N:P, N:S, N:Ca, N:K, N:Mg, N:Mn, N:Zn) (see Supplementary Table 5).

A majority of the elements were more abundant in galleries of *X. saxesenii*, except Ca, which was found in higher concentrations in galleries of *T. lineatum* (cf. y-axes in Figures 6–9). This is striking, because some element ratios like C:P (36061.25 vs. 29225.61), N:P (20.41 vs. 8.3), N:S (10.93 vs. 6.22), N:Ca (8.76 vs. 1.5) and N:Zn (294.49 vs. 57.22) were much smaller in beech control xylem of *X. saxesenii* than spruce control xylem of *T. lineatum*. This is only partly explainable by the higher C:N ratio of spruce control xylem (3523.37 vs. 1766.57) (see Supplementary Table 5).

## Discussion

Fungus-farming ambrosia beetles breed in xylem which is extremely poor in many essential nutrients and very much biased toward C, H, and O (Fengel and Wegener, 1989). It has been a long-standing question how the nutritional needs of the beetles are satisfied within this extreme habitat. Here, we show that the galleries of three, phylogenetically unrelated ambrosia beetles are enriched by several essential elements, out of which the enrichment ratios relative to xylem are most striking for P, S, and N. For example, the *Raffaelea* fungal mutualists of *X. saxesenii* are apparently able to greatly enrich their host’s galleries with P by a factor of 16.4 relative to beech control xylem with a molar C:P ratio of only 36061.25, indicating high availability of P for the beetles.

Detailed measurements revealed that there is a sharp increase in elemental composition where the gallery borders the xylem (see Figure 2 and Figure 4). It appears that the mutualistic, filamentous ambrosia fungi are translocating and concentrating essential elements from the surrounding xylem to their fruiting structures (i.e., conidiophores and conidia) on the gallery walls, which are then available to their beetle hosts as food. While N might be additionally enriched by bacterial fixation from the atmosphere (Bridges, 1981; Six, 2013), galleries are aerobic and make this possibility unlikely. No other elements are fixed from the atmosphere and thus translocation from outside of the gallery (from the xylem) would be required for such increases as we observed to occur. Translocation from the more nutrient-rich phloem is also, in principal, possible, but may be excluded by the finding that the enrichment of elements was generally lower in entrance tunnels of the galleries closer to the phloem of the colonized trees (see Supplementary Figure 2). Yeasts and bacteria, which are ubiquitous associates within ambrosia beetle galleries (Beaver, 1989; Davis, 2015; Kirkendall et al., 2015; Hulcr and Stelinski, 2017), are unicellular organisms and thus are not capable of growing into and translocating elements from the xylem to the gallery, so we propose that enrichment is solely done by the (ambrosia) fungal symbionts.

If translocation of nutrients from the immediate surrounding of galleries by the mutualistic fungi occurs, we should find a depletion of elements near galleries but not in areas distant to galleries where fungi have not colonized the xylem. However, there was no detectable difference in elemental composition of xylem surrounding the galleries (assumed to be colonized by fungi) and control xylem located some distance from the galleries (assumed to be uncolonized) (Figures 6–9). Even though the enrichment ratio of the surrounding xylem relative to the control xylem was in a few cases >1, the enrichment was not statistically significant for any micronutrient (Figures 6–9; see also molar ratios in Supplementary Table 5). Qualitatively this finding was also supported by similar elemental compositions of presumably fungus-colonized vs. uncolonized xylem samples taken in vertical vs. horizontal directions (with and against the grain) using SEM-EDX (Figures 1–5 and Supplementary Figures 1–3). Only H was significantly more abundant in surrounding xylem of *T. lineatum* galleries, which might indicate enzymatic cleavage of H-bonds by the *Phialophoropsis* fungal mutualists that are known to be potent degraders of xylan and cellulose (Lehenberger et al., 2019).

We have a few potential explanations why we did not detect such a pattern: First, ambrosia beetles (or their symbionts) may acquire nutrients independent of the xylem. However, apart from some symbiotic bacteria that might fix atmospheric N (Peklo and Satava, 1949; Bridges, 1981; Morales-Jiménez et al., 2009; Six, 2013), none of the other elements are fixed from the atmosphere and galleries are isolated from the external environment so we regard this explanation rather unlikely. Second, it may be that the fungi colonize much deeper into the xylem than previously thought. Given the low concentration of the elements in xylem (Figures 6–9, but see also Pretzsch et al., 2014), the fungi may need to colonize large areas of the xylem or even access more nutrient-rich parts of trees like phloem (Pretzsch et al., 2014; Ulbricht et al., 2016). Also, this option we regard rather unlikely because ambrosia beetle fungi are generally thought to be rather poor wood decomposers and may grow only a few mm inside the xylem (Francke-Grosmann, 1966). An exception may be *Phialophoropsis ferruginea* (associated with *T. lineatum*), which is depolymerizing major wood compounds such as cellulose and xylan (Lehenberger et al., 2019) and can grow up to 20 cm inside the xylem (Francke-Grosmann, 1966). But also for *T. lineatum*, there was no indication for stronger enrichment of the surrounding xylem. Regarding the translocation from phloem we did not see stronger enrichments of elements near the phloem in *X. saxesenii* entrance tunnels (see above). Third, xylem serves as an elemental pipeline between roots and leaves (and *vice versa*) and within living trees elements (in low concentrations) are constantly moving within the xylem (Matyssek et al., 2010). As ambrosia beetles are colonizing weakened or recently dead trees (Kirkendall et al., 2015), there might be still enough flux of elements through the xylem so that fungi might not necessarily need to colonize large areas of the xylem, but may capture essential elements out of the sap flow in the immediate surrounding of their galleries. It is questionable, however, if there is still enough flux or diffusion of elements in sap in wind-thrown or cut timber, which is also colonized by these beetles. Future studies on the fungal growth within the xylem are needed to answer whether the fungi, sap flow or diffusion are moving the elements toward the vicinity of the galleries.

The quantification analysis revealed striking differences regarding the concentration of certain elements between the galleries of *X. saxesenii* and *T. lineatum*. Generally, galleries of *X. saxesenii* contained much higher amounts of K, Mg, Mn, P, S, and N and the availability of especially N and P (based on molar ratios of C:P and C:N, see Supplementary Table 5) is much better than in galleries of *T. lineatum*. The only element that was found in higher amounts within the galleries of *T. lineatum* was Ca (N:Ca ratio of gallery: *T. lineatum* = 1.5, *X. saxesenii* = 8.76), which is known to affect the growth, branching, sporulation and/or virulence in fungi (Pitt and Ugalde, 1984; Lange and Peiter, 2020). Partly this is explainable by the higher abundances of some elements in the xylem of beech (i.e., control xylem from *X. saxesenii*; Figures 6, 7; Supplementary Table 5) than in the xylem of spruce (i.e., control xylem from *T. lineatum*; Figures 8, 9; Supplementary Table 5), with Ca being the only exception (∼350 ppm in beech vs. ∼550 ppm in spruce) (see also Pretzsch et al., 2014; Ulbricht et al., 2016). On the other hand, also elemental enrichment is strongest in *X. saxesenii*, followed by *T. lineatum* and then *E. dermestoides* (as we used a semi-quantitative pattern, we can only assume that this might be the case for *E. dermestoides* based on the findings of the SEM-EDX analysis and the use of only one gallery). Interestingly, this pattern correlates with the social behavior and productivity (in terms of offspring numbers per gallery) of these species. Sociality is a derived trait in ambrosia beetles and cooperative breeding and eusociality are found in very few species (Kirkendall et al., 2015; Biedermann and Vega, 2020). The higher social species produce longer galleries and produce more offspring. Hence, more individuals spend longer periods inside the tree allowing more time for the fungi to move elements to their structures growing on gallery walls. *X. saxesenii* can occupy the same gallery with dozens of individuals over multiple generations for up to 2 years, whereas the other two species have only a single generation of a dozen individuals per gallery which lasts for only a few months (*T. lineatum*) or a single individual that develops over 2 years (*E. dermestoides*) (Francke-Grosmann, 1952, 1967, 1975; Popo and Thalenhorst, 1974; Biedermann and Taborsky, 2011; Biedermann et al., 2013).

Wood is finite in N, P, and other essential elements, which may also mean that the *Raffaelea* fungi of this *X. saxesenii* are particularly efficient to capture elements or may extend deeper into xylem than others (see option 2 or 3 above). This could, on the other hand, also be the precondition for *X. saxesenii* to evolve a social life with overlapping generations and long-term productivity. More work is needed to determine whether the stronger elemental enrichment in *X. saxesenii* is the cause or consequence of/for its social life. First, we need to know how deeply ambrosia fungi forage in xylem and whether different species have different foraging efficiencies that feedback to influence host fecundity. One important question that remains to be answered is whether different host trees influence elemental enrichment and beetle fitness. It would be interesting to investigate if the ambrosia fungi of *X. saxesenii* are able to provide equally high levels of enrichment when foraging within the less nutritional xylem of spruce and how any differences may translate to effects on beetle fitness and sociality.

Apart from the questions raised above it would be interesting to investigate elemental accumulation from fungi growing in the galleries to the adult beetles and larvae (e.g., Six and Elser, 2020), which could give us insights into the specialization of ambrosia beetles to dead wood habitat. Furthermore, we need to determine how these elements may cycle within the gallery between beetles and their symbionts. In *X. saxesenii*, adults and larvae have been shown to press their feces against gallery walls, possibly to be recycled and further degraded by their fungal mutualists (De Fine Licht and Biedermann, 2012). Furthermore, there is unpublished data that uric acid (the major excretory product of insects) is one of the best nitrogen sources in media for culturing *Raffaelea sulphurea*, one of the two mutualists of *X. saxesenii* [R. Roeper, unpublished data; or see Norris (1979) for *Fusarium* ambrosia fungi].

Besides bark and ambrosia beetles, also fungus-farming ants and termites may benefit from their associated mutualistic fungi in terms of nutrient cycling and thus element availability. Despite the low number of stoichiometric studies on these insects, there are hints for similar mechanisms. For instance, Li et al. (2012) found, that certain elements are enriched within the gut system of the wood-feeding termite *O. formosanus* as well as within its symbiotic fungus and the surrounding nest soil. As suggested by the authors, certain endo- and ectosymbiotic microbes might be involved in this pattern. Moreover, a recent experimental study on this species showed that fungal combs (macerated woody material colonized by a fungus) colonized by the symbiotic *Termitomyces* fungus, is highly enriched with several elements compared to combs without the *Termitomyces* species, indicating a potential role of this fungus in element translocation and concentration (see Yang et al., 2020). It would be interesting to examine and compare the main groups of fungus-farming insects (attine ants, termites, ambrosia beetles) and, in particular, the abilities of their mutualistic microbes to translocate and concentrate elements from provisioned plant biomass.

In ambrosia beetles, stable isotopes could be used to observe the nutrient-flow from woody tissue and fungi to the larvae and beetles (e.g., Hobbie et al., 1999; Griffith, 2004; Mayor et al., 2009). In the natural substrate this will be difficult, but a few ambrosia beetle species (e.g., *X. saxesenii*) can be reared within artificial media that may be supplemented with isotopes of interest (Gries and Sanders, 1984; Biedermann et al., 2009). Ecological stoichiometry is still in its infancy in insect-fungus mutualisms, but its application could help us to identify the nutrient flows upon which these systems are based. This may not only help us to understand their nutritional requirements and mechanisms of element capture but might also help us to identify the key microbial players in these typically multipartite systems. Ultimately, ES studies conducted across a broad range of Scolytinae will allow us to develop a holistic model for the evolution of these mutualisms in what is arguably the most important group of insects affecting forest ecosystems worldwide.

## Data Availability Statement

The original contributions presented in the study are included in the article/Supplementary Material, further inquiries can be directed to the corresponding author.

## Author Contributions

ML, DS, and PB designed the study and wrote the manuscript (the latest with input of AG and NF). ML and PB collected galleries of studied ambrosia beetles and prepared samples for the further process. Samples for SEM-EDX were prepared by ML, while ML and NF did the SEM-EDX analysis. AG did the elemental quantification of samples. ML analyzed data, did statistics, calculated ratios, and plotted results. All authors contributed to the article and approved the submitted version.

## Conflict of Interest

The authors declare that the research was conducted in the absence of any commercial or financial relationships that could be construed as a potential conflict of interest.

## Supplementary Material

Supplementary Figure 1 Elemental composition of gallery walls and xylem surrounding a gallery of *Xyleborinus saxesenii* (ID = X.sax2) using SEM-EDX. The gallery was examined in vertical **(A,B)** and horizontal **(C,D)** direction respective to wood grain; location and numbers of measured areas are given in the photos on the right. The *Y*-axis of each plot is counts per second (cps) per electronvolt (eV) of each element, while the *X*-axis is the applied electricity in kilo-electronvolt (keV).

Supplementary Figure 2 Elemental composition of gallery walls, entrance tunnel and xylem surrounding a gallery of *Xyleborinus saxesenii* (ID = X.sax3) using SEM-EDX. The gallery was examined in vertical **(A,B)** and horizontal **(C–E)** direction respective to wood grain; location and numbers of measured areas are given in the photos on the right. The *Y*-axis of each plots is counts per second (cps) per electronvolt (eV) of each element, while the *X*-axis is showing the applied electricity in kilo-electronvolt (keV).

Supplementary Figure 3 Elemental composition of gallery walls, pupal chambers and xylem surrounding a gallery of *Trypodendron lineatum* (ID = T.lin2) using SEM-EDX. Pupal chambers and surrounding xylem were examined in vertical **(A–C)** direction. The gallery was examined in horizontal **(D,E)** direction; location and numbers of measured areas are given in the photos on the right. The *Y*-axis of each plots is counts per second (cps) per electronvolt (eV) of each element, while the *X*-axis is the applied electricity in kilo-electronvolt (keV).
