# Supplementary material for: Nutrient-Poor Breeding Substrates of Ambrosia Beetles Are Enriched With Biologically Important Elements
Source: Front Microbiol. 2021 Apr 26;12:664542. doi: 10.3389/fmicb.2021.664542 (PMC8107399; doi:10.3389/fmicb.2021.664542)
Supplement: Supplementary file 1 [file Table_1.DOCX]

Supplementary Material

**Table S1:** Overview of replicates (N) per element and treatment, and statistical differences between the three types of samples for *Trypodendron lineatum*: ns - p > 0.05, * - p < 0.05, ** - p < 0.01, *** - p < 0.001.

| **Element** | **Treatment** | **Number of replicates (N)** | **Tested combination** | **Statistics (p)** |
| --- | --- | --- | --- | --- |
| Ca | Gallery | 8 | Gallery vs. Surrounding xylem | **0.0281 (*)** |
| Ca | Surrounding xylem | 8 | Gallery vs. Control xylem | **0.0004 (***)** |
| Ca | Control xylem | 8 | Surrounding xylem vs. Control xylem | 0.1662 (ns) |
| Cu | Gallery | 8 | Gallery vs. Surrounding xylem | 0.0756 (ns) |
| Cu | Surrounding xylem | 8 | Gallery vs. Control xylem | 0.0667 (ns) |
| Cu | Control xylem | 8 | Surrounding xylem vs. Control xylem | 0.9690 (ns) |
| Fe | Gallery | 8 | Gallery vs. Surrounding xylem | 0.5086 (ns) |
| Fe | Surrounding xylem | 8 | Gallery vs. Control xylem | 08914 (ns) |
| Fe | Control xylem | 8 | Surrounding xylem vs. Control xylem | 0.7837 (ns) |
| K | Gallery | 8 | Gallery vs. Surrounding xylem | 0.1855 (ns) |
| K | Surrounding xylem | 8 | Gallery vs. Control xylem | **0.0149 (*)** |
| K | Control xylem | 8 | Surrounding xylem vs. Control xylem | 0.4337 (ns) |
| Mg | Gallery | 8 | Gallery vs. Surrounding xylem | **0.0479 (*)** |
| Mg | Surrounding xylem | 8 | Gallery vs. Control xylem | **0.0026 (**)** |
| Mg | Control xylem | 8 | Surrounding xylem vs. Control xylem | 0.4134 (ns) |
| Mn | Gallery | 7 | Gallery vs. Surrounding xylem | 1.0 (ns) |
| Mn | Surrounding xylem | 7 | Gallery vs. Control xylem | 0.3056 (ns) |
| Mn | Control xylem | 7 | Surrounding xylem vs. Control xylem | 0.3066 (ns) |
| P | Gallery | 7 | Gallery vs. Surrounding xylem | **<.0001 (***)** |
| P | Surrounding xylem | 8 | Gallery vs. Control xylem | **<.0001 (***)** |
| P | Control xylem | 8 | Surrounding xylem vs. Control xylem | 0.9709 (ns) |
| S | Gallery | 7 | Gallery vs. Surrounding xylem | **<.0001 (***)** |
| S | Surrounding xylem | 8 | Gallery vs. Control xylem | **<.0001 (***)** |
| S | Control xylem | 8 | Surrounding xylem vs. Control xylem | 0.6843 (ns) |
| Zn | Gallery | 8 | Gallery vs. Surrounding xylem | 0.4810 (ns) |
| Zn | Surrounding xylem | 8 | Gallery vs. Control xylem | 0.1353 (ns) |
| Zn | Control xylem | 8 | Surrounding xylem vs. Control xylem | 0.6875 (ns) |
| C | Gallery | 6 | Gallery vs. Surrounding xylem | 0.5705 (ns) |
| C | Surrounding xylem | 8 | Gallery vs. Control xylem | 0.7993 (ns) |
| C | Control xylem | 8 | Surrounding xylem vs. Control xylem | 0.1963 (ns) |
| H | Gallery | 6 | Gallery vs. Surrounding xylem | 0.9385 (ns) |
| H | Surrounding xylem | 7 | Gallery vs. Control xylem | **0.0003 (***)** |
| H | Control xylem | 7 | Surrounding xylem vs. Control xylem | **0.0004 (***)** |
| N | Gallery | 5 | Gallery vs. Surrounding xylem | **0.0005 (***)** |
| N | Surrounding xylem | 8 | Gallery vs. Control xylem | **0.0036 (**)** |
| N | Control xylem | 7 | Surrounding xylem vs. Control xylem | 0.6177 (ns) |

**Table S2:** Overview of replicates (N) per element and treatment, and statistical differences between the three types of samples for *Xyleborinus saxesenii*: ns - p > 0.05, * - p < 0.05, ** - p < 0.01, *** - p < 0.001.

| **Element** | **Treatment** | **Number of replicates (N)** | **Tested combination** | **Statistics (p)** |
| --- | --- | --- | --- | --- |
| Ca | Gallery | 9 | Gallery vs. Surrounding xylem | **0.0005 (***)** |
| Ca | Surrounding xylem | 9 | Gallery vs. Control xylem | **0.0141 (*)** |
| Ca | Control xylem | 10 | Surrounding xylem vs. Control xylem | 0.3609 (ns) |
| Cu | Gallery | 10 | Gallery vs. Surrounding xylem | 0.2482 (ns) |
| Cu | Surrounding xylem | 10 | Gallery vs. Control xylem | 0.9985 (ns) |
| Cu | Control xylem | 9 | Surrounding xylem vs. Control xylem | 0.2445 (ns) |
| Fe | Gallery | 10 | Gallery vs. Surrounding xylem | 0.8158 (ns) |
| Fe | Surrounding xylem | 10 | Gallery vs. Control xylem | 0.9986 (ns) |
| Fe | Control xylem | 10 | Surrounding xylem vs. Control xylem | 0.8428 (ns) |
| K | Gallery | 10 | Gallery vs. Surrounding xylem | **<.0001 (***)** |
| K | Surrounding xylem | 10 | Gallery vs. Control xylem | **<.0001 (***)** |
| K | Control xylem | 10 | Surrounding xylem vs. Control xylem | 0.9946 (ns) |
| Mg | Gallery | 9 | Gallery vs. Surrounding xylem | **<.0001 (***)** |
| Mg | Surrounding xylem | 10 | Gallery vs. Control xylem | **<.0001 (***)** |
| Mg | Control xylem | 10 | Surrounding xylem vs. Control xylem | 0.7659 (ns) |
| Mn | Gallery | 9 | Gallery vs. Surrounding xylem | **0.0066 (**)** |
| Mn | Surrounding xylem | 9 | Gallery vs. Control xylem | **0.0087 (**)** |
| Mn | Control xylem | 10 | Surrounding xylem vs. Control xylem | 0.6907 (ns) |
| P | Gallery | 10 | Gallery vs. Surrounding xylem | **<.0001 (***)** |
| P | Surrounding xylem | 10 | Gallery vs. Control xylem | **<.0001 (***)** |
| P | Control xylem | 10 | Surrounding xylem vs. Control xylem | 0.1976 (ns) |
| S | Gallery | 10 | Gallery vs. Surrounding xylem | **<.0001 (***)** |
| S | Surrounding xylem | 10 | Gallery vs. Control xylem | **<.0001 (***)** |
| S | Control xylem | 10 | Surrounding xylem vs. Control xylem | 0.9990 (ns) |
| Zn | Gallery | 10 | Gallery vs. Surrounding xylem | **0.0173 (*)** |
| Zn | Surrounding xylem | 9 | Gallery vs. Control xylem | 0.0928 (ns) |
| Zn | Control xylem | 10 | Surrounding xylem vs. Control xylem | 0.6875 (ns) |
| C | Gallery | 10 | Gallery vs. Surrounding xylem | 0.6734 (ns) |
| C | Surrounding xylem | 10 | Gallery vs. Control xylem | 0.3893 (ns) |
| C | Control xylem | 10 | Surrounding xylem vs. Control xylem | 0.8814 (ns) |
| H | Gallery | 10 | Gallery vs. Surrounding xylem | 0.9892 (ns) |
| H | Surrounding xylem | 9 | Gallery vs. Control xylem | 0.8712 (ns) |
| H | Control xylem | 9 | Surrounding xylem vs. Control xylem | 0.8076 (ns) |
| N | Gallery | 10 | Gallery vs. Surrounding xylem | **<.0001 (***)** |
| N | Surrounding xylem | 10 | Gallery vs. Control xylem | **<.0001 (***)** |
| N | Control xylem | 10 | Surrounding xylem vs. Control xylem | 0.8040 (ns) |

**Table S3:** Enrichment ratios (ER) for each examined element based on the relative proportion of the means of the two treatments “Gallery” and “Surrounding xylem” to “Control xylem” for the samples of *Trypodendron lineatum*.

| **Element** | **Treatment** | **ER** |
| --- | --- | --- |
| Ca | Gallery | 1.73 |
| Ca | Surrounding xylem | 1.27 |
| Cu | Gallery | 1.87 |
| Cu | Surrounding xylem | 0.61 |
| Fe | Gallery | 0.86 |
| Fe | Surrounding xylem | 1.16 |
| K | Gallery | 1.68 |
| K | Surrounding xylem | 1.26 |
| Mg | Gallery | 1.7 |
| Mg | Surrounding xylem | 1.22 |
| Mn | Gallery | 2.5 |
| Mn | Surrounding xylem | 2.32 |
| P | Gallery | 3.54 |
| P | Surrounding xylem | 1.02 |
| S | Gallery | 1.91 |
| S | Surrounding xylem | 0.88 |
| Zn | Gallery | 1.38 |
| Zn | Surrounding xylem | 1.17 |
| C | Gallery | 0.99 |
| C | Surrounding xylem | 0.99 |
| H | Gallery | 1.17 |
| H | Surrounding xylem | 1.15 |
| N | Gallery | 3.09 |
| N | Surrounding xylem | 0.8 |

**Table S4:** Enrichment ratios (ER) for each examined element based on the relative proportion of the means of the two treatments “Gallery” and “Surrounding xylem” to “Control xylem” for the samples of *Xyleborinus saxesenii*.

| **Element** | **Treatment** | **ER** |
| --- | --- | --- |
| Ca | Gallery | 1.41 |
| Ca | Surrounding xylem | 0.91 |
| Cu | Gallery | 0.97 |
| Cu | Surrounding xylem | 0.67 |
| Fe | Gallery | 0.96 |
| Fe | Surrounding xylem | 0.91 |
| K | Gallery | 2.19 |
| K | Surrounding xylem | 1.02 |
| Mg | Gallery | 1.7 |
| Mg | Surrounding xylem | 0.93 |
| Mn | Gallery | 1.48 |
| Mn | Surrounding xylem | 1.01 |
| P | Gallery | 16.4 |
| P | Surrounding xylem | 1.8 |
| S | Gallery | 3.31 |
| S | Surrounding xylem | 1.01 |
| Zn | Gallery | 1.47 |
| Zn | Surrounding xylem | 0.85 |
| C | Gallery | 1 |
| C | Surrounding xylem | 1 |
| H | Gallery | 1 |
| H | Surrounding xylem | 1 |
| N | Gallery | 4.27 |
| N | Surrounding xylem | 1.28 |

**Table S5:** Carbon:phosphorus (C:P), C:nitrogen (N), N:P, N:sulfur (S), N:Ca (calcium), N:potassium (K), N:Mg (magnesium), N:Mn (manganese), and N:Zn (zinc) ratios (molar, as different elements were compared) for each of the three types of samples of *Trypodendron lineatum* and *Xyleborinus saxesenii*.

| *Trypodendron lineatum* | | *Xyleborinus saxesenii* | |
| --- | --- | --- | --- |
| C:P Control | 29225.61 | C:P Control | 36061.25 |
| C:P Gallery | 8231.68 | C:P Gallery | 2210.59 |
| C:P Surrounding xylem | 28539.64 | C:P Surrounding xylem | 20066.83 |
| C:N Control | 3523.37 | C:N Control | 1766.57 |
| C:N Gallery | 1135.91 | C:N Gallery | 416.24 |
| C:N Surrounding xylem | 4412.99 | C:N Surrounding xylem | 1385.25 |
| N:P Control | 8.3 | N:P Control | 20.41 |
| N:P Gallery | 10.08 | N:P Gallery | 5.31 |
| N:P Surrounding xylem | 6.47 | N:P Surrounding xylem | 14.49 |
| N:S Control | 6.22 | N:S Control | 10.93 |
| N:S Gallery | 10.08 | N:S Gallery | 14.06 |
| N:S Surrounding xylem | 5.64 | N:S Surrounding xylem | 13.85 |
| N:Ca Control | 0.84 | N:Ca Control | 2.91 |
| N:Ca Gallery | 1.5 | N:Ca Gallery | 8.76 |
| N:Ca Surrounding xylem | 0.53 | N:Ca Surrounding xylem | 4.07 |
| N:K Control | 1.21 | N:K Control | 1.09 |
| N:K Gallery | 2.23 | N:K Gallery | 2.12 |
| N:K Surrounding xylem | 0.77 | N:K Surrounding xylem | 1.37 |
| N:Mg Control | 3.85 | N:Mg Control | 3.7 |
| N:Mg Gallery | 6.96 | N:Mg Gallery | 9.28 |
| N:Mg Surrounding xylem | 2.52 | N:Mg Surrounding xylem | 5.08 |
| N:Mn Control | 190.73 | N:Mn Control | 8.85 |
| N:Mn Gallery | 236.06 | N:Mn Gallery | 26.18 |
| N:Mn Surrounding xylem | 65.29 | N:Mn Surrounding xylem | 11.48 |
| N:Zn Control | 84.32 | N:Zn Control | 196.32 |
| N:Zn Gallery | 188.89 | N:Zn Gallery | 569.33 |
| N:Zn Surrounding xylem | 57.22 | N:Zn Surrounding xylem | 294.49 |
