# Supplementary material for: Nutrient-Poor Breeding Substrates of Ambrosia Beetles Are Enriched With Biologically Important Elements
Source: Front Microbiol. 2021 Apr 26;12:664542. doi: 10.3389/fmicb.2021.664542 (PMC8107399; doi:10.3389/fmicb.2021.664542)
Supplement: Supplementary file 5 [file Data_Sheet_4.docx]

*Trypodendron lineatum* – Output of the statistical analysis

Linear models with sqrt transformed data

Signif. codes: 0 ‘***’ 0.001 ‘**’ 0.01 ‘*’ 0.05 ‘.’ 0.1 ‘ ’ 1

**Ca**

$`lsmeans`

Sample lsmean SE df lower.CL upper.CL

Control xylem 22.8 1.13 21 20.4 25.1

Gallery 30.3 1.13 21 27.9 32.7

Surrounding xylem 25.8 1.13 21 23.5 28.2

Results are given on the sqrt (not the response) scale.

Confidence level used: 0.95

$contrasts

contrast estimate SE df t.ratio p.value

Control xylem - Gallery -7.51 1.6 21 -4.685 0.0004

Control xylem - Surrounding xylem -3.03 1.6 21 -1.891 0.1661

Gallery - Surrounding xylem 4.48 1.6 21 2.794 0.0281

**Cu**

$`lsmeans`

Sample lsmean SE df lower.CL upper.CL

Control xylem 0.888 0.261 21 0.345 1.43

Gallery 1.829 0.261 21 1.287 2.37

Surrounding xylem 0.976 0.261 21 0.434 1.52

Results are given on the sqrt (not the response) scale.

Confidence level used: 0.95

$contrasts

contrast estimate SE df t.ratio p.value

Control xylem - Gallery -0.9418 0.369 21 -2.554 0.0667

Control xylem - Surrounding xylem -0.0882 0.369 21 -0.239 0.9690

Gallery - Surrounding xylem 0.8536 0.369 21 2.315 0.0756

**Fe**

$`lsmeans`

Sample lsmean SE df lower.CL upper.CL

Control xylem 3.81 0.376 21 3.03 4.59

Gallery 3.57 0.376 21 2.79 4.35

Surrounding xylem 4.17 0.376 21 3.38 4.95

Results are given on the sqrt (not the response) scale.

Confidence level used: 0.95

$contrasts

contrast estimate SE df t.ratio p.value

Control xylem - Gallery 0.243 0.532 21 0.458 0.8914

Control xylem - Surrounding xylem -0.356 0.532 21 -0.669 0.7837

Gallery - Surrounding xylem -0.599 0.532 21 -1.127 0.5086

**K**

$`lsmeans`

Sample lsmean SE df lower.CL upper.CL

Control xylem 18.6 1.32 21 15.9 21.4

Gallery 24.4 1.32 21 21.6 27.1

Surrounding xylem 21.0 1.32 21 18.2 23.7

Results are given on the sqrt (not the response) scale.

Confidence level used: 0.95

$contrasts

contrast estimate SE df t.ratio p.value

Control xylem - Gallery -5.77 1.87 21 -3.084 0.0149

Control xylem - Surrounding xylem -2.35 1.87 21 -1.258 0.4337

Gallery - Surrounding xylem 3.41 1.87 21 1.826 0.1855

**Mg**

$`lsmeans`

Sample lsmean SE df lower.CL upper.CL

Control xylem 8.32 0.471 21 7.34 9.3

Gallery 10.88 0.471 21 9.89 11.9

Surrounding xylem 9.18 0.471 21 8.20 10.2

Results are given on the sqrt (not the response) scale.

Confidence level used: 0.95

$contrasts

contrast estimate SE df t.ratio p.value

Control xylem - Gallery -2.557 0.666 21 -3.837 0.0026

Control xylem - Surrounding xylem -0.863 0.666 21 -1.295 0.4134

Gallery - Surrounding xylem 1.694 0.666 21 2.542 0.0479

**Mn**

> lsmeans( object=m6, pairwise ~ Sample, adjust= "tukey")

Note: Use 'contrast(regrid(object), ...)' to obtain contrasts of back-transformed estimates

$`lsmeans`

Sample lsmean SE df lower.CL upper.CL

Control xylem 1.14 0.579 18 -0.0766 2.36

Gallery 2.38 0.579 18 1.1671 3.60

Surrounding xylem 2.38 0.579 18 1.1653 3.60

Results are given on the sqrt (not the response) scale.

Confidence level used: 0.95

$contrasts

contrast estimate SE df t.ratio p.value

Control xylem - Gallery -1.24368 0.818 18 -1.520 0.3056

Control xylem - Surrounding xylem -1.24184 0.818 18 -1.517 0.3066

Gallery - Surrounding xylem 0.00184 0.818 18 0.002 1.0000

**P**

$`lsmeans`

Sample lsmean SE df lower.CL upper.CL

Control xylem 6.22 0.528 20 5.12 7.32

Gallery 12.10 0.564 20 10.92 13.28

Surrounding xylem 6.39 0.528 20 5.29 7.49

Results are given on the sqrt (not the response) scale.

Confidence level used: 0.95

$contrasts

contrast estimate SE df t.ratio p.value

Control xylem - Gallery -5.882 0.773 20 -7.612 <.0001

Control xylem - Surrounding xylem -0.173 0.746 20 -0.232 0.9709

Gallery - Surrounding xylem 5.709 0.773 20 7.388 <.0001

**S**

$`lsmeans`

Sample lsmean SE df lower.CL upper.CL

Control xylem 7.48 0.332 20 6.79 8.17

Gallery 10.46 0.355 20 9.72 11.20

Surrounding xylem 7.09 0.332 20 6.39 7.78

Results are given on the sqrt (not the response) scale.

Confidence level used: 0.95

$contrasts

contrast estimate SE df t.ratio p.value

Control xylem - Gallery -2.976 0.486 20 -6.128 <.0001

Control xylem - Surrounding xylem 0.393 0.469 20 0.838 0.6843

Gallery - Surrounding xylem 3.369 0.486 20 6.938 <.0001

**Zn**

$`lsmeans`

Sample lsmean SE df lower.CL upper.CL

Control xylem 2.89 0.194 21 2.49 3.29

Gallery 3.44 0.194 21 3.04 3.84

Surrounding xylem 3.12 0.194 21 2.72 3.52

Results are given on the sqrt (not the response) scale.

Confidence level used: 0.95

$contrasts

contrast estimate SE df t.ratio p.value

Control xylem - Gallery -0.550 0.274 21 -2.007 0.1353

Control xylem - Surrounding xylem -0.228 0.274 21 -0.832 0.6875

Gallery - Surrounding xylem 0.322 0.274 21 1.174 0.4810

**C H N Profiling**

**H**

$`lsmeans`

Sample lsmean SE df lower.CL upper.CL

Control xylem 2.58 0.0278 18 2.52 2.64

Gallery 2.79 0.0321 18 2.72 2.86

Surrounding xylem 2.78 0.0297 18 2.71 2.84

Results are given on the sqrt (not the response) scale.

Confidence level used: 0.95

$contrasts

contrast estimate SE df t.ratio p.value

Control xylem - Gallery -0.2119 0.0424 18 -4.992 0.0003

Control xylem - Surrounding xylem -0.1971 0.0407 18 -4.844 0.0004

Gallery - Surrounding xylem 0.0149 0.0437 18 0.340 0.9385

**N**

$`lsmeans`

Sample lsmean SE df lower.CL upper.CL

Control xylem 0.171 0.0231 17 0.1220 0.220

Gallery 0.308 0.0274 17 0.2504 0.366

Surrounding xylem 0.141 0.0216 17 0.0951 0.186

Results are given on the sqrt (not the response) scale.

Confidence level used: 0.95

$contrasts

contrast estimate SE df t.ratio p.value

Control xylem - Gallery -0.1373 0.0358 17 -3.833 0.0036

Control xylem - Surrounding xylem 0.0301 0.0317 17 0.949 0.6177

Gallery - Surrounding xylem 0.1674 0.0349 17 4.798 0.0005

**C**

$`lsmeans`

Sample lsmean SE df lower.CL upper.CL

Control xylem 6.869 0.008557 19 6.851 6.887

Gallery 6.861 0.009881 19 6.841 6.882

Surrounding xylem 6.858 0.008557 19 6.840 6.876

Results are given on the sqrt (not the response) scale.

Confidence level used: 0.95

$contrasts

contrast estimate SE df t.ratio p.value

Control xylem - Gallery 0.00790 0.0131 19 0.605 0.8194

Control xylem - Surrounding xylem 0.01105 0.0121 19 0.913 0.6389

Gallery - Surrounding xylem 0.00315 0.0131 19 0.241 0.9686

*Xyleborinus saxesenii* – Output of the statistical analysis

Linear models with sqrt transformed data

Signif. codes: 0 ‘***’ 0.001 ‘**’ 0.01 ‘*’ 0.05 ‘.’ 0.1 ‘ ’ 1

**Ca**

$`lsmeans`

Sample lsmean SE df lower.CL upper.CL

Control xylem 17.8 0.649 26 16.4 19.1

Gallery 20.6 0.684 26 19.2 22.0

Surrounding xylem 16.5 0.649 26 15.1 17.8

Results are given on the sqrt (not the response) scale.

Confidence level used: 0.95

$contrasts

contrast estimate SE df t.ratio p.value

Control xylem - Gallery -2.87 0.943 26 -3.044 0.0141

Control xylem - Surrounding xylem 1.28 0.918 26 1.390 0.3609

Gallery - Surrounding xylem 4.15 0.943 26 4.397 0.0005

**Cu**

$`lsmeans`

Sample lsmean SE df lower.CL upper.CL

Control xylem 1.52 0.147 26 1.222 1.83

Gallery 1.51 0.139 26 1.227 1.80

Surrounding xylem 1.19 0.139 26 0.904 1.48

Results are given on the sqrt (not the response) scale.

Confidence level used: 0.95

$contrasts

contrast estimate SE df t.ratio p.value

Control xylem - Gallery 0.0107 0.203 26 0.053 0.9985

Control xylem - Surrounding xylem 0.3338 0.203 26 1.647 0.2445

Gallery - Surrounding xylem 0.3230 0.197 26 1.638 0.2482

**Fe**

$`lsmeans`

Sample lsmean SE df lower.CL upper.CL

Control xylem 3.33 0.312 27 2.69 3.96

Gallery 3.35 0.312 27 2.71 3.99

Surrounding xylem 3.08 0.312 27 2.44 3.72

Results are given on the sqrt (not the response) scale.

Confidence level used: 0.95

$contrasts

contrast estimate SE df t.ratio p.value

Control xylem - Gallery -0.0226 0.441 27 -0.051 0.9986

Control xylem - Surrounding xylem 0.2464 0.441 27 0.559 0.8428

Gallery - Surrounding xylem 0.2689 0.441 27 0.610 0.8158

**K**

$`lsmeans`

Sample lsmean SE df lower.CL upper.CL

Control xylem 27.9 1.14 27 25.6 30.2

Gallery 41.5 1.14 27 39.2 43.9

Surrounding xylem 28.1 1.14 27 25.7 30.4

Results are given on the sqrt (not the response) scale.

Confidence level used: 0.95

$contrasts

contrast estimate SE df t.ratio p.value

Control xylem - Gallery -13.632 1.61 27 -8.477 <.0001

Control xylem - Surrounding xylem -0.159 1.61 27 -0.099 0.9946

Gallery - Surrounding xylem 13.473 1.61 27 8.379 <.0001

**Mg**

$`lsmeans`

Sample lsmean SE df lower.CL upper.CL

Control xylem 12.0 0.433 26 11.1 12.9

Gallery 15.6 0.457 26 14.7 16.5

Surrounding xylem 11.5 0.433 26 10.7 12.4

Results are given on the sqrt (not the response) scale.

Confidence level used: 0.95

$contrasts

contrast estimate SE df t.ratio p.value

Control xylem - Gallery -3.616 0.630 26 -5.742 <.0001

Control xylem - Surrounding xylem 0.429 0.613 26 0.700 0.7659

Gallery - Surrounding xylem 4.045 0.630 26 6.423 <.0001

**Mn**

$`lsmeans`

Sample lsmean SE df lower.CL upper.CL

Control xylem 11.6 0.490 25 10.6 12.6

Gallery 13.9 0.516 25 12.9 15.0

Surrounding xylem 11.5 0.516 25 10.4 12.5

Results are given on the sqrt (not the response) scale.

Confidence level used: 0.95

$contrasts

contrast estimate SE df t.ratio p.value

Control xylem - Gallery -2.319 0.711 25 -3.260 0.0087

Control xylem - Surrounding xylem 0.144 0.711 25 0.203 0.9776

Gallery - Surrounding xylem 2.464 0.730 25 3.375 0.0066

**P**

$`lsmeans`

Sample lsmean SE df lower.CL upper.CL

Control xylem 5.55 0.734 27 4.05 7.06

Gallery 23.26 0.734 27 21.75 24.76

Surrounding xylem 7.39 0.734 27 5.89 8.90

Results are given on the sqrt (not the response) scale.

Confidence level used: 0.95

$contrasts

contrast estimate SE df t.ratio p.value

Control xylem - Gallery -17.70 1.04 27 -17.060 <.0001

Control xylem - Surrounding xylem -1.84 1.04 27 -1.773 0.1976

Gallery - Surrounding xylem 15.86 1.04 27 15.287 <.0001

**S**

$`lsmeans`

Sample lsmean SE df lower.CL upper.CL

Control xylem 8.02 0.307 27 7.39 8.65

Gallery 14.57 0.307 27 13.94 15.20

Surrounding xylem 8.04 0.307 27 7.41 8.67

Results are given on the sqrt (not the response) scale.

Confidence level used: 0.95

$contrasts

contrast estimate SE df t.ratio p.value

Control xylem - Gallery -6.5445 0.435 27 -15.058 <.0001

Control xylem - Surrounding xylem -0.0186 0.435 27 -0.043 0.9990

Gallery - Surrounding xylem 6.5260 0.435 27 15.015 <.0001

**Zn**

`

Sample lsmean SE df lower.CL upper.CL

Control xylem 2.65 0.188 26 2.27 3.04

Gallery 3.23 0.188 26 2.85 3.62

Surrounding xylem 2.43 0.198 26 2.02 2.83

Results are given on the sqrt (not the response) scale.

Confidence level used: 0.95

$contrasts

contrast estimate SE df t.ratio p.value

Control xylem - Gallery -0.580 0.266 26 -2.185 0.0928

Control xylem - Surrounding xylem 0.227 0.273 26 0.831 0.6875

Gallery - Surrounding xylem 0.807 0.273 26 2.957 0.0173

**C H N Profiling**

**C**

$`lsmeans`

Sample lsmean SE df lower.CL upper.CL

Control xylem 6.834 0.008579 27 6.816 6.851

Gallery 6.850 0.008579 27 6.832 6.868

Surrounding xylem 6.840 0.008579 27 6.822 6.857

Results are given on the sqrt (not the response) scale.

Confidence level used: 0.95

$contrasts

contrast estimate SE df t.ratio p.value

Control xylem - Gallery -0.01618 0.0121 27 -1.334 0.3893

Control xylem - Surrounding xylem -0.00582 0.0121 27 -0.480 0.8814

Gallery - Surrounding xylem 0.01036 0.0121 27 0.854 0.6734

**H**

$`lsmeans`

Sample lsmean SE df lower.CL upper.CL

Control xylem 2.50 0.0121 25 2.47 2.52

Gallery 2.51 0.0115 25 2.48 2.53

Surrounding xylem 2.51 0.0121 25 2.48 2.53

Results are given on the sqrt (not the response) scale.

Confidence level used: 0.95

$contrasts

contrast estimate SE df t.ratio p.value

Control xylem - Gallery -0.00837 0.0167 25 -0.502 0.8712

Control xylem - Surrounding xylem -0.01071 0.0171 25 -0.626 0.8076

Gallery - Surrounding xylem -0.00234 0.0167 25 -0.140 0.9892

**N**

$`lsmeans`

Sample lsmean SE df lower.CL upper.CL

Control xylem 0.246 0.0271 27 0.191 0.302

Gallery 0.500 0.0271 27 0.444 0.555

Surrounding xylem 0.270 0.0271 27 0.215 0.326

Results are given on the sqrt (not the response) scale.

Confidence level used: 0.95

$contrasts

contrast estimate SE df t.ratio p.value

Control xylem - Gallery -0.2534 0.0383 27 -6.609 <.0001

Control xylem - Surrounding xylem -0.0242 0.0383 27 -0.632 0.8040

Gallery - Surrounding xylem 0.2292 0.0383 27 5.978 <.0001
