# Supplementary figures and images for: Nutrient-Poor Breeding Substrates of Ambrosia Beetles Are Enriched With Biologically Important Elements
Source: Front Microbiol. 2021 Apr 26;12:664542. doi: 10.3389/fmicb.2021.664542 (PMC8107399; doi:10.3389/fmicb.2021.664542)

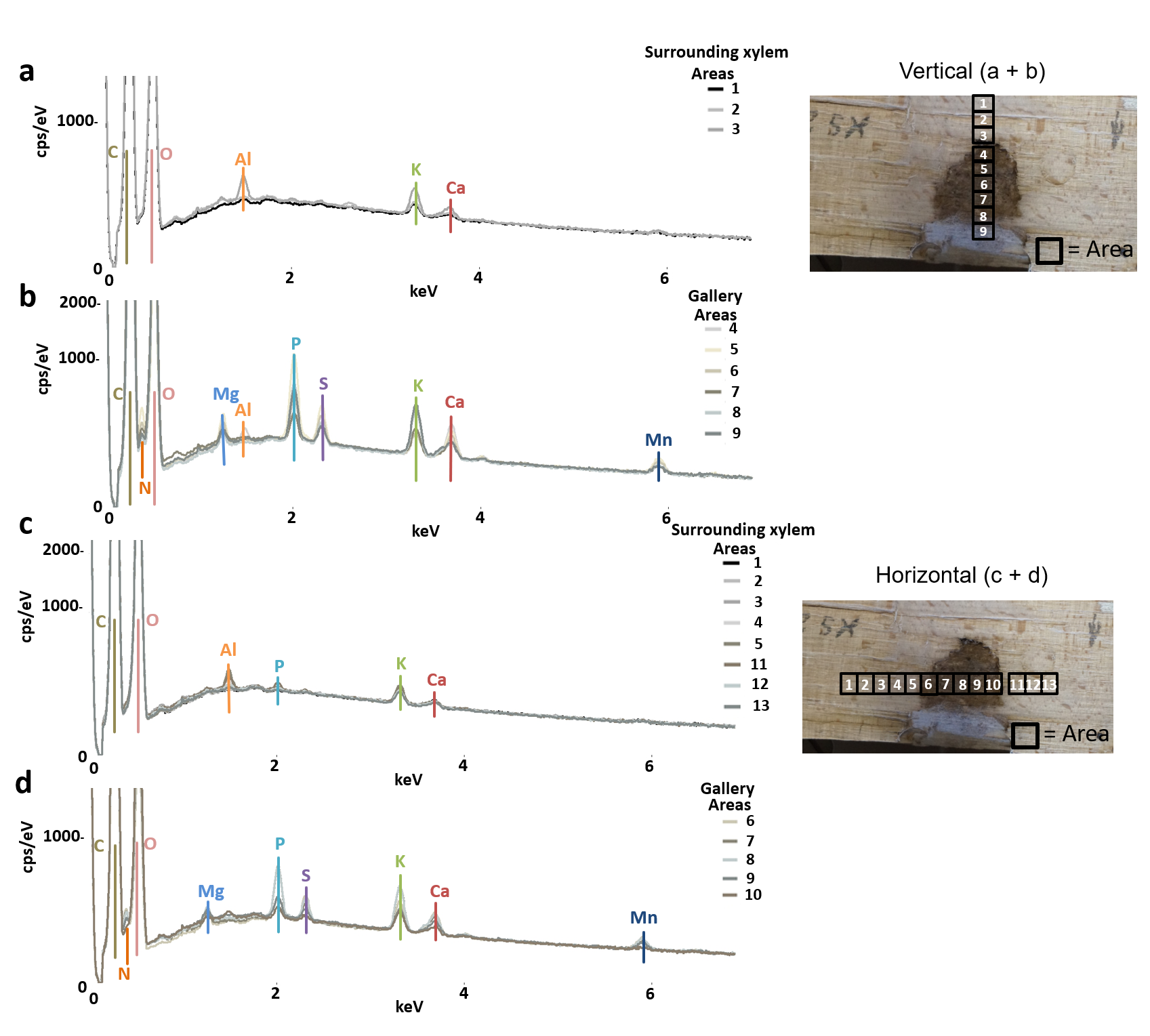

Supplement: Supplementary Figure 1 — Elemental composition of gallery walls and xylem surrounding a gallery of Xyleborinus saxesenii (ID = X.sax2) using SEM-EDX. The gallery was examined in vertical (A,B) and horizontal (C,D) direction respective to wood grain; location and numbers of measured areas are given in the photos on the right. The Y-axis of each plot is counts per second (cps) per electronvolt (eV) of each element, while the X-axis is the applied electricity in kilo-electronvolt (keV). [file Image_1.TIF]

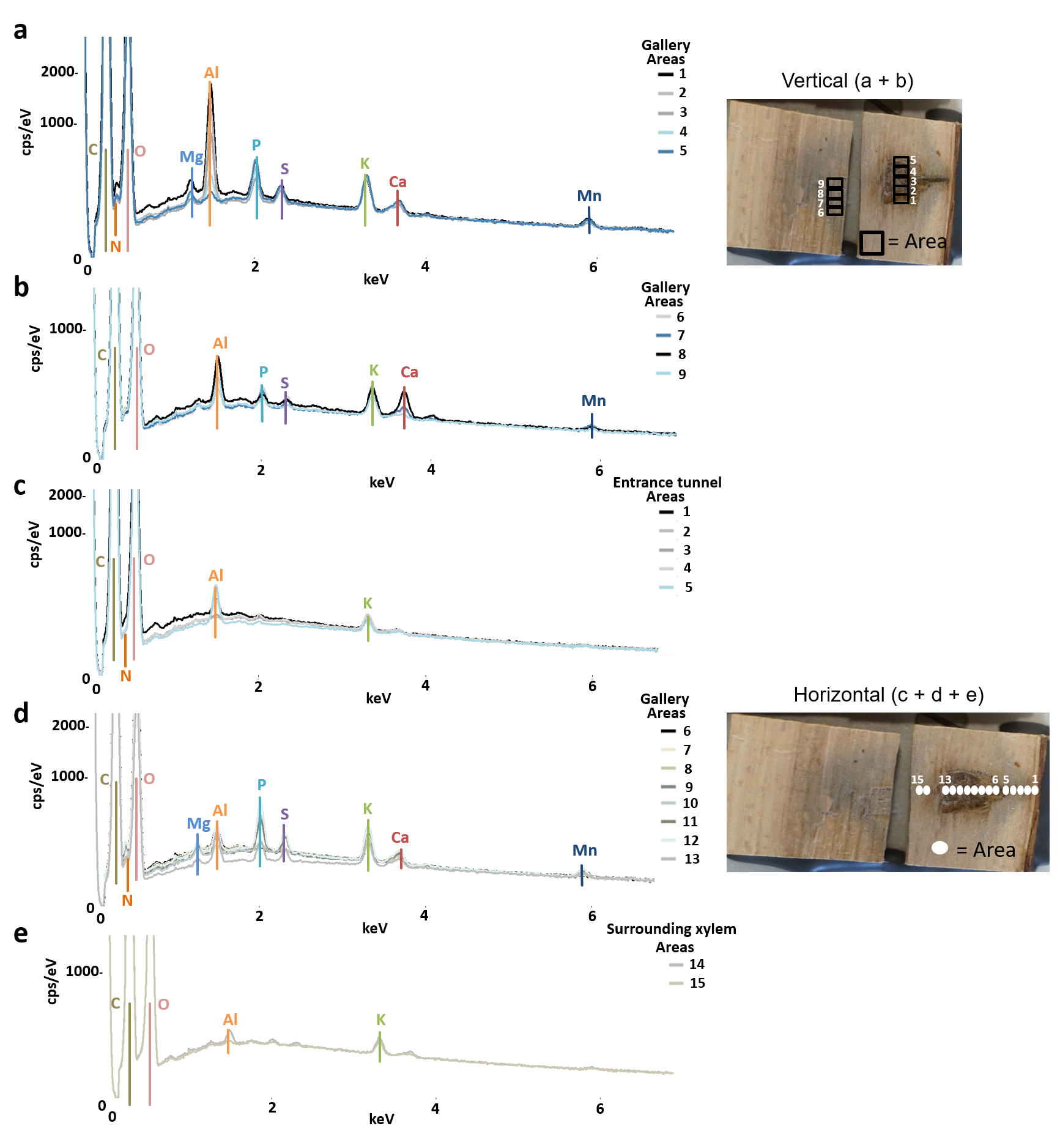

Supplement: Supplementary Figure 2 — Elemental composition of gallery walls, entrance tunnel and xylem surrounding a gallery of Xyleborinus saxesenii (ID = X.sax3) using SEM-EDX. The gallery was examined in vertical (A,B) and horizontal (C–E) direction respective to wood grain; location and numbers of measured areas are given in the photos on the right. The Y-axis of each plots is counts per second (cps) per electronvolt (eV) of each element, while the X-axis is showing the applied electricity in kilo-electronvolt (keV). [file Image_2.TIF]

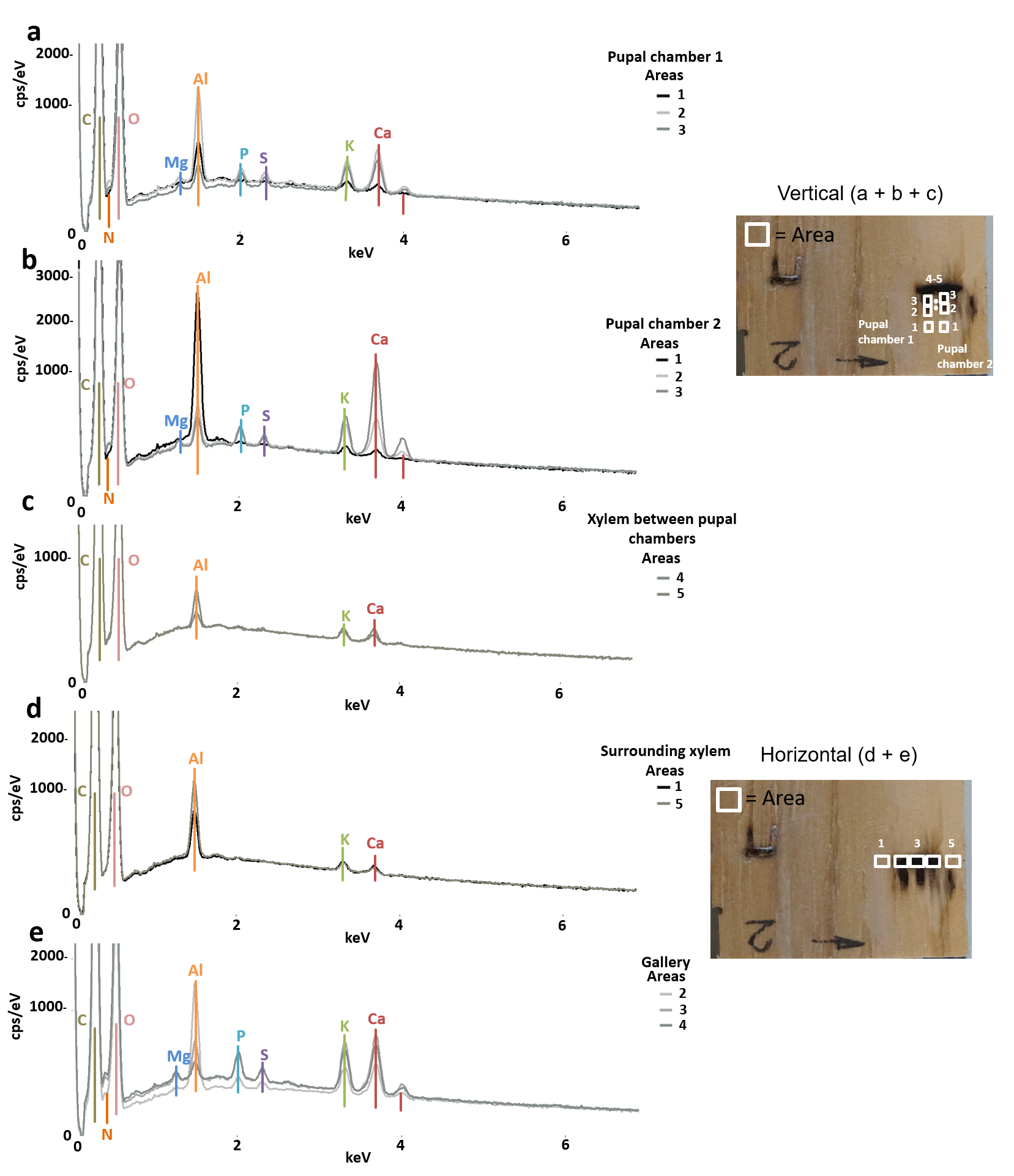

Supplement: Supplementary Figure 3 — Elemental composition of gallery walls, pupal chambers and xylem surrounding a gallery of Trypodendron lineatum (ID = T.lin2) using SEM-EDX. Pupal chambers and surrounding xylem were examined in vertical (A–C) direction. The gallery was examined in horizontal (D,E) direction; location and numbers of measured areas are given in the photos on the right. The Y-axis of each plots is counts per second (cps) per electronvolt (eV) of each element, while the X-axis is the applied electricity in kilo-electronvolt (keV). [file Image_3.TIF]
